# Micelles as Delivery Vehicles for Oligofluorene for Bioimaging

**DOI:** 10.1371/journal.pone.0024425

**Published:** 2011-09-06

**Authors:** Fengyu Su, Ruhaniyah Alam, Qian Mei, Yanqing Tian, Deirdre R. Meldrum

**Affiliations:** Center for Biosignatures Discovery Automation, Biodesign Institute, Arizona State University, Tempe, Arizona, United States of America; RMIT University, Australia

## Abstract

With the successful development of organic/polymeric light emitting diodes, many organic and polymeric fluorophores with high quantum efficiencies and optical stability were synthesized. However, most of these materials which have excellent optical properties are insoluble in water, limiting their applications in biological fields. Herein, we used micelles formed from an amino-group-containing poly(*ε*-caprolactone)-*block*-poly(ethylene glycol) (PCL-*b*-PEG-NH_2_) to incorporate a hydrophobic blue emitter oligofluorene (**OF**) to enable its application in biological conditions. Although **OF** is completely insoluble in water, it was successfully transferred into aqueous solutions with a good retention of its photophysical properties. **OF** exhibited a high quantum efficiency of 0.84 in a typical organic solvent of tetrahydrofuran (THF). In addition, **OF** also showed a good quantum efficiency of 0.46 after being encapsulated into micelles. Two cells lines, human glioblastoma (U87MG) and esophagus premalignant (CP-A), were used to study the cellular internalization of the **OF** incorporated micelles. Results showed that the hydrophobic **OF** was located in the cytoplasm, which was confirmed by co-staining the cells with nucleic acid specific SYTO 9, lysosome specific LysoTracker Red®, and mitochondria specific MitoTracker Red. MTT assay indicated non-toxicity of the **OF**-incorporated micelles. This study will broaden the application of hydrophobic functional organic compounds, oligomers, and polymers with good optical properties to enable their applications in biological research fields.

## Introduction

Fluorescence bioimaging, labeling, and detection require fluorescence probes for studying complex and dynamic cellular processes [1]. The current widely studied and applied probes can be classified as (1) organic molecular probes including fluorescein derivatives [1], rhodamines [1] and cyanine dyes [1] and (2) inorganic probes including quantum dots (QD) [1] and up-conversion nanoparticles [2]–[4]. Traditional organic molecular probes usually exhibit poor photostability when applied in long-term monitoring of live cells. The inorganic probes such as QDs are considered as alternative probes due to their excellent optical properties, such as high photostability, narrow emission, and high brightness. However, the inherent toxicity of QDs mainly from the heavy metal cores with cadmium ions is a significant concern for long-term bioapplications [5]–[6]. Also the QDs require suitable surface modification to enable their applications in biological fields. Due to these setbacks, there is a growing need for the development of new fluorescent probes.

On the other hand, along with the successful development of organic and polymeric materials for light emitting diodes (OLED and PLED), many highly fluorescent organic compounds, oligomers, and polymers having better photostability than the traditional organic fluorescent probes were synthesized [7], [8]. However, many of these materials are insoluble in water, limiting their bioapplications. In general, three approaches have been utilized to enable their applications in biological fields. The first approach is a chemical modification of compounds/polymers with water soluble segments or moieties [9]–[15]; the second approach is the preparation of organic/polymer nanoparticles through a polymer precipitation approach [16]–[20]; the third way utilizes micelles formed from amphiphilic block copolymers to encapsulate hydrophobic organic molecules and polymers to enable their applications in biological environments [21]–[25].

We have significant interests in the third method, which uses micelles formed from amphiphilic block copolymers to deliver hydrophobic conjugated polymers. Using this approach, conjugated polymers can be hydrophobic, endowing their easy preparation and purification. Micelles are biocompatible and have long blood circulation time. In addition, their surfaces can be modified with suitable targeting moieties for targeted delivery and imaging. Up to date, the most popularly studied hydrophobic conjugated polymers are poly[9,9-dihexylfluorene-*alt*-9,9-bis(2-(2-(2-methoxyethoxy)ethoxy)ethyl)fluorene] (PF), poly[9,9-bis(2-(2-(2-methoxyethoxy)ethoxy)ethyl)-fluorenyldivinylene-*alt*-9,9-bis(3-tbutylpropanoate) fluorene] (PFV), poly[9,9-bis(2-(2-(2-methoxyethoxy)ethoxy)ethyl)fluorene-*alt*-4,7-(2,1,3-benzothiadiazol)] (PFBT), poly[2-methoxy-5-(2′-ethyl-hexyloxy)-1,4-phenylenevinylene] (MEH-PPV), and poly[2-(2′,5′-bis-(2″-ethylhexyloxy)phenyl)-1,4-phenylene vinylene] (BEHP-PPV) with various polymeric molecular weights [21]-[25]. Micelles used to achieve the delivery are made from block copolymers such as: 2-diacyl-*sn*-glycero-3-phosphoethanolamine-*N*-[methoxy(polyethylene glycol)-2000] (PEG2000-PE), 1,2-dipalmitoyl-*sn*-glycero-3-phosphocholine (DPPC), polystyrene-*random*-PEG-grafted-polystyrene with carboxylic groups (PS-PEG-COOH), and poly(DL-lactide-*co*-glycolide) (PLGA) [21]–[25]. Typical cell lines explored are breast cancer MCF-7, breast cancer SKBR-3, neuroblastoma SHSY-5Y, human epidermoid cancer Hep-2, and fibroblast NIH 3T3 [21]–[25].

**Figure 1:**
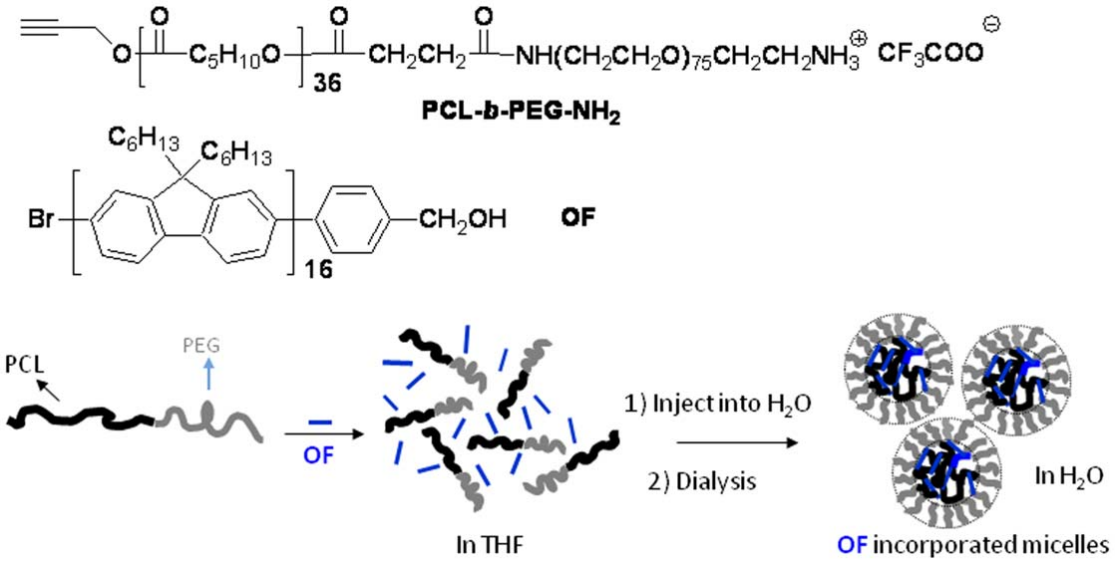
Chemical structures of PCL-*b*-PEG-NH_2_ and OF and the schematic drawing of incorporation of hydrophobic OF using PCL-*b*-PEG-NH_2_.

Previously, we used polymeric micelles to encapsulate hydrophobic two-photon absorbing materials to achieve high two-photon absorbing cross-sections in aqueous solutions [26], to enhance singlet oxygen generation efficiency of the photosensitizer [27], and to use the hydrophobic materials as probes for bioimaging in mouse macrophages [28]. Along with the line of our study, herein, we use an oligofluorene (**OF**, Figure 1) as a model hydrophobic conjugated probe for bioimging through an encapsulation of **OF** into micelles of an amphiphilic block copolymer of an amine-containing poly(*ε*-caprolactone)-*block*-poly(ethylene glycol) (PCL-*b*-PEG-NH_2_). Photophysical properties of **OF** in the micelles in addition to the subcellular locations and cytotoxicity of the **OF**-encapsulated micelles to two cell lines, human glioblastoma U87MG and esophageal premalignant CP-A, were investigated.

## Materials and Methods

### Materials

The amine-containing block copolymer of PCL-*b*-PEG-NH_2_ (*M*_n_ = 13300, *M*_w_ = 17200, *M*_w_/*M*_n_ = 1.29) and **OF** (*M*_n_ = 5800, *M*_w_ = 9300, *M*_w_/*M*_n_ = 1.61) were prepared according to published procedures [28], [29]. Dialysis membranes (regenerate cellulose, *M*_w_ cut off 10000) were purchased from Pierce (Rockford, IL). Eagle's Minimum Essential Medium (EMEM) was ordered from ATCC (Manassas, VA). Keratinocyte medium, SYTO 9, LysoTracker Red®, and MitoTracker Red were acquired from Invitrogen (Carlsbad, CA). EMEM medium was used for U87MG cell culture and Keratinocyte medium was used for CP-A cell culture.

### General methods

Dynamic light scattering (DLS) measurements for micelle diameters were performed using a Malvern Nano-ZS instrument equipped with a 4 mW He-Ne laser (633 nm) with an output at a scattering angle of 173°. Solution was passed through a 0.45 µm Nylon micro-filter (VWR, Batavia, IL) to remove dust before the DLS measurements. A Shimadzu UV-3600 UV-Vis-NIR spectrophotometer (Shimadzu Scientific Instruments, Columbia, MD) was used for absorption spectra measurements. A Shimadzu RF-5301 spectrofluorophotometer was used for fluorescence measurements.

### Preparation of micelles

5.8 mg of **OF** was dissolved in 1 mL tetrahydrofuran (THF) to make a 1 mM stock solution. 25 mg of the block copolymer of PCL-*b*-PEG-NH_2_ was added into the solution. 200 µL of the mixture was taken out and dispersed slowly into 1 mL of water using a syringe with a 22½ gauge needle to generate micelles. Dialysis was performed on the micelles using a regenerated cellulose dialysis membrane (MWCO = 10,000) in 10 mM 4-(2-hydroxyethyl)-1-piperazineethanesulfonic acid (HEPES, pH 7.2) buffer to remove THF. Buffer was changed twice per day for three days. Micellar solution was taken out from the dialysis bag and was filtered using a 0.22 µm microfilter to remove any possible large particles. Concentration of **OF** in micelles was determined to be 120 µM using UV-Vis spectrophotometer (detailed procedure was given in supplementary materials of Text S1 and a Figure S1). Its corresponding concentration of PCL-*b*-PEG-NH_2_ was 3 mg/mL.

### Release study of OF from micelles

Release kinetics of **OF** from the micelles was investigated at 37°C by dialysis. Briefly, 3 mL of each micelle-based nanocarrier, loaded as previously described, was placed in a dialysis bag. Dialysis was performed against 0.3 L of 10 mM HEPES solutions. 25 µL of micelle solution was drawn at time intervals from the nanocarrier dispersion, further diluted with DMSO, and then used to determine **OF** concentration with a UV-Vis spectrophotometer. Release percentage, or retention percentage in an opposite way, was calculated based on the absorbance change.

### Determination of quantum yields

Fluorescence quantum yields (*η)* of samples in solutions were recorded by using quinine sulfate in 1.0 M H_2_SO_4_ (*η* = 0.55, excitation wavelength of 365 nm) [30] according to the following equation [31]:
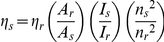
where *η*_r_ and *η*_s_ are the fluorescence quantum yields of the standard and the samples, respectively. *A*_r_ and *A*_s_ are the absorbance of the standard and the measured samples at the excitation wavelength, respectively. *I*_r_ and *I*_s_ are the integrated emission intensities of the standard and the samples, respectively. *n*_r_ and *n*_s_ are the refractive indices of the corresponding solvents of the solutions, respectively. The experimental error was ∼10%.

### Cell culture and imaging

U87MG cells (American Type Culture Collection, ATCC, Manassas, VA) were cultured in EMEM supplemented with 10% fetal bovine serum, 100 u/mL of penicillin, 2 mM L-glutamine (Sigma-Aldrich), and incubated at 37°C in a 5% CO_2_ atmosphere. CP-A cells (kindly provided by Dr. Brian J. Reid at Fred Hutchison Cancer Research Center, Seattle, WA) were cultured in Keratinocyte-serum free medium (Invitrogen, Carlsbad, CA) supplemented with Bovine Pituitary Extract (BPE) and human recombinant Epidermal Growth Factor (rEGF, Invitrogen) at 37°C in a 5% CO_2_ atmosphere. Cells were seeded onto 96-well plates at 10,000 cells per well, and incubated for one day. **OF** loaded micelles made from PCL-*b*-PEG-NH_2_ were dissolved and diluted in EMEM growth medium to give final **OF** concentrations of 2–10 µM and polymer concentrations of 0.05–0.25 mg/mL for cellular internalization.

To confirm micelles' subcellular distributions, nuclei acid staining SYTO 9, lysosome and late endosome specific LysoTracker Red®, and mitochondria specific MitoTracker Red were used to co-stain cells with micelles. Cells were first internalized with micelles overnight. The medium was then removed and cells were washed with fresh medium. Then SYTO 9, LyoTracker Red®, or MitoTracker Red were added separately. Cells were then incubated for 20 minutes before imaging. Concentrations of SYTO 9, LysoTracker Red®, and MitoTracker Red were 100 nM each. Under Nikon Eclipse TE2000E confocal fluorescence microscope (Melville, NY), **OF** was excited at 402 nm and its blue emission was collected using a 450/35 nm filter set; SYTO 9 was excited at 488 nm and its green emission was collected using a 515/30 nm filter set; LysoTracker Red® was excited at 561 nm and its red emission was collected using a 605/75 nm filter set; MitoTracker Red was excited at 561 nm and its red emission was collected using a 605/75 nm filter set. Negligible background fluorescence of cells was detected under the setting used.

### Cytotoxicity study

The assay was performed by using an *in vitro* MTT based toxicology assay kit (Promega, Madison, WI). Cells incubated with **OF** loaded micelles for 24 hours in 96-well plate were washed with PBS buffer and then incubated in fresh medium (100 µL) and 15 µL of MTT solution (5 mg/mL) in 5% CO_2_ at 37°C for another 3 hours. 100 µL of Solubilization Solution/Stop Mix (Promega) was added to each well to dissolve the internalized purple formazan crystals by gentle pipette movements. Absorbance of formazan was measured at 570 nm using SpectraMax 190 from Molecular Devices (Downingtown, PA). Each experiment was conducted in triplicate. Results were expressed as percentages of the absorbance of blank controls without micelles.

## Results and Discussion

### Micelle preparation and stability studies

**Figure 2:**
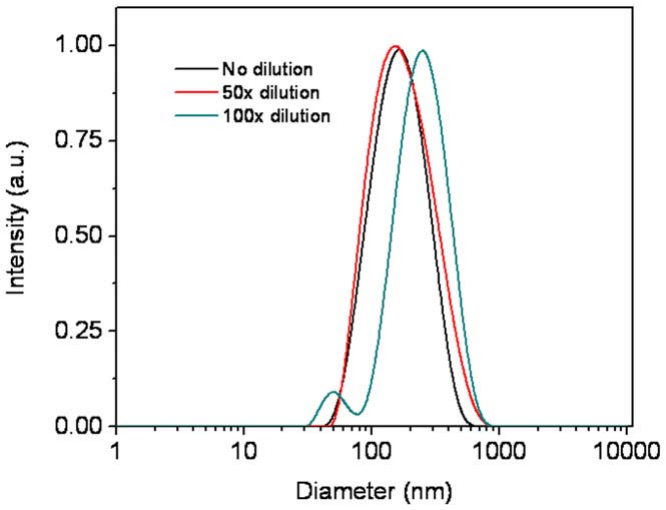
DLS of the OF-incorporated micelles.

**Figure 3:**
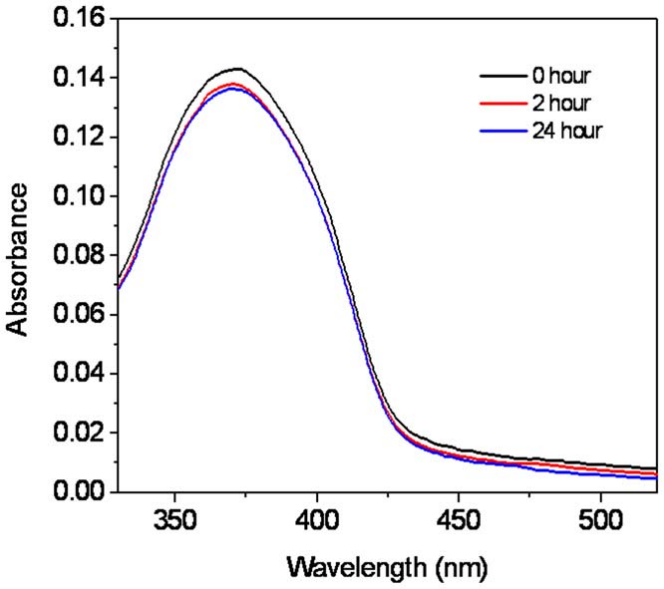
Time dependent absorbance change of the OF-incorporated micelles in HEPES buffer at 37°C.

Although **OF** is completely insoluble in water, it can be incorporated into the micelles formed from block copolymers (Figure 1), which allows the hydrophobic oligomer to be used in an aqueous solution. For the PEG-*b*-PCL-NH_2_ type block copolymers, the PCL segment acts as the hydrophobic core and the hydrophilic PEG segment functions as the shell. The core of the micelle (PCL) can function as a reservoir to accommodate the hydrophobic material. The shell (PEG), having a brush-like protective corona, can ensure a complete dispersion of the micelles in water. Micelles were prepared using a dialysis approach in 10 mM HEPES buffer solutions and characterized using DLS, showing that the micelles had an average diameter of 140 nm (Figure 2). The micelles have good stability during their storage at 4°C for at least one month, without any size or photophysical properties' changing. Stability was also studied by dilution of the micelles at room temperature. Dilution to 50 fold of the micelles did not change average sizes of the micelles. 100 Fold dilution of the micelles resulted in a reorganization and disassembly of the micelles, which can be seen by the observation of the larger and smaller bimodal sizes (Figure 2). Therefore, for all the bioimaging studies, the micelles were diluted to less than 50 fold. Release of **OF** from micelles was studied at 37°C against HEPES buffer. After 24 hours releasing test at 37°C, less than 5% **OF** was released from the micelles (Figure 3), suggesting sufficient stability of the micelles in a biological environment.

### Photophysical properties

**Figure 4:**
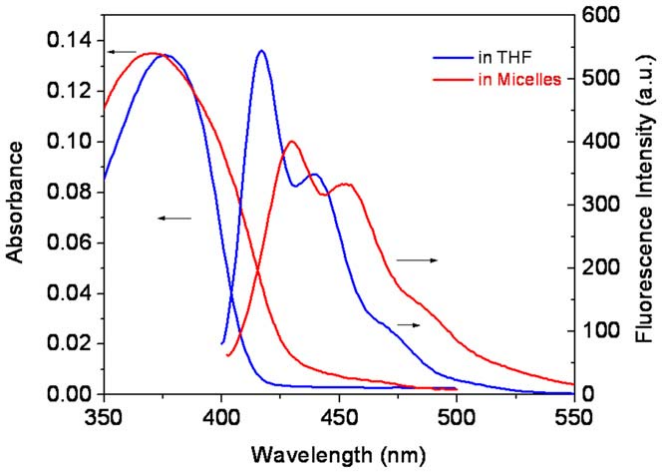
Photophysical properties of OF in THF and in micelles.

Photophysical properties of **OF** were investigated in organic solvent (THF) and micellar aqueous solution (Figure 4). In THF, **OF** exhibits an absorption maximum at 377 nm. In micelles, the absorption becomes much broader and the maximum is slightly blue-shifted to 371 nm. Fluorescence spectrum in the micelles is also different from that in THF, where a significant red shift was observed. The relative intensity ratios of the first (417 nm for **OF** in THF, 430 nm for **OF** in micelles) and the second (441 nm for **OF** in THF, 453 nm for **OF** in micelles) vibrational bands in the emission spectra are different. In water, the polymers form micelles with **OF** as the core, resulting in much stronger aggregations of the **OF** chains due to enhanced π-π interactions or hydrophobic-hydrophobic interactions of the **OF** segments in the micellar cores. Because of the strong aggregations of **OF** segments in the micellar cores, a blue-shifted effect was observed from the absorption of **OF** in micelles. The blue shifted absorption indicates H-aggregations [32] of **OF** moieties in the micelles, which resulted in a red-shifted emission and decrease of the quantum efficiency. Quantum yield of **OF** segments in the micelles decreased to 0.46 from 0.84 in THF. It should be noted here, **OF** is completely insoluble in water. However, using the micelle approach, **OF** can be incorporated into micelles and applied in aqueous solutions with a retention of reasonably good photophysical properties.

### Imaging and cytotoxicity

**Figure 5:**
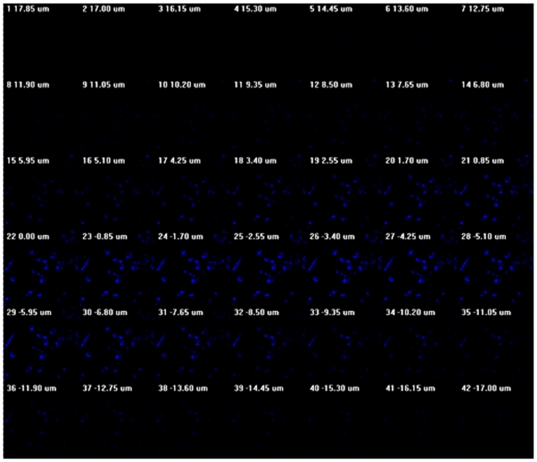
Z-stack of the OF/PCL-*b*-PEG-NH_2_ micelles in CP-A cells.

Cellular uptake of micelles containing **OF** by U87MG and CP-A cells was studied by using confocal laser scanning microscopy. Cells were incubated with **OF**-containing micelles first. After cellular internalization for 24 hours, blue emissions were observed under confocal fluorescence microscope, showing the cellular uptake of the micelles. Considering the high stability of micelles, we believe the whole micelles were taken up by cells. In order to confirm whether micelles were located intracellularly or on the membrane, 42 z slices (Figure 5) were acquired at a sampling distance of 35 µm along the z-axis using a 60× oil objective. Clear distance dependent fluorescence intensity was observed, indicating the micelles had successfully crossed cell membrane and were located inside cells.

In order to further understand the subcellular locations of **OF** incorporated micelles, SYTO 9, LysoTracker Red®, and MitoTracker Red were used to costain the cells. In general, Hoechst and DAPI are popularly used as nucleus-selective dyes. However, these two types of dyes exhibit blue emissions, which have interferences with the blue emissions from **OF**. Therefore, SYTO 9, a green emitter which stains nucleic acids was used as a replacement of the Hoechst or DAPI. It should be noted that SYTO 9 dye does not act exclusively as a nuclear stain in live cells. However, the emission intensity from the stained nuclei, especially the nucleolus, is much stronger than those of other organelles.

**Figure 6:**
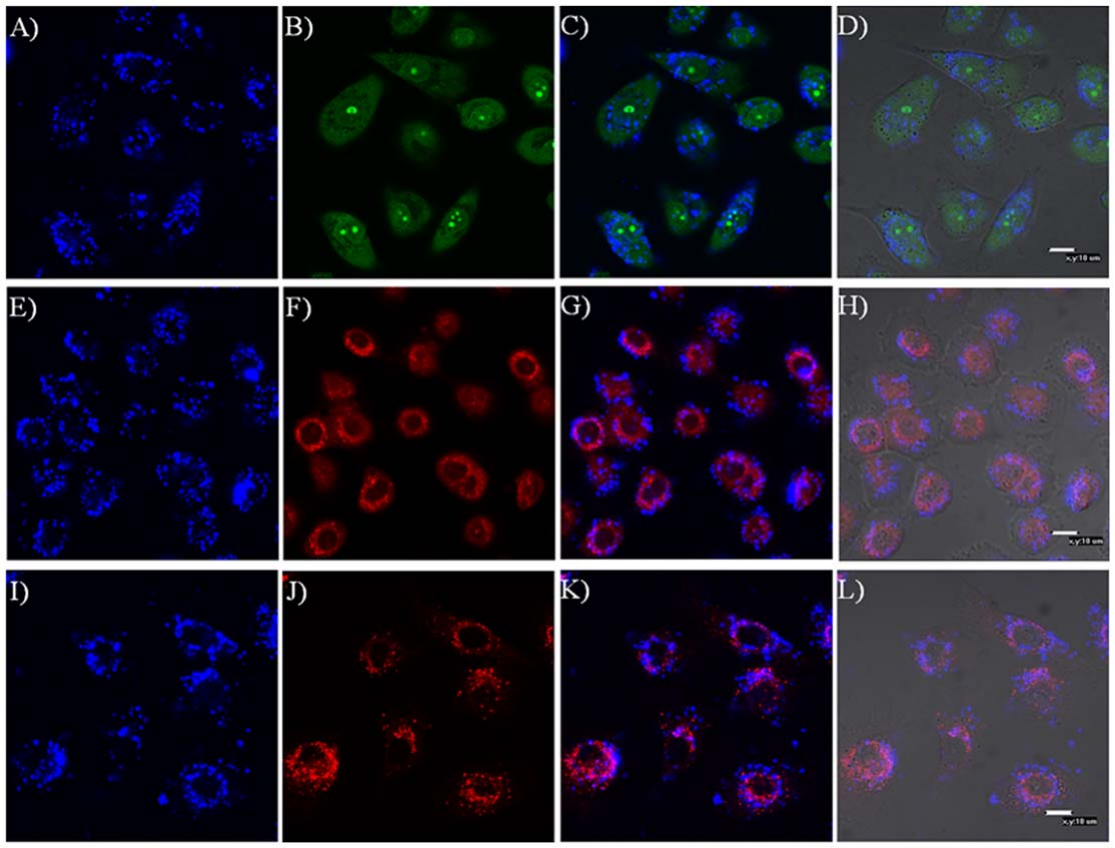
Confocal fluorescence microscopy images of the OF/PCL-*b*-PEG-NH_2_ micelles for CP-A cells. A, E and I: fluorescence images of micelles; B: fluorescence image of SYTO 9; C: overlay of A and B; D: overlay of C with bright field image; F: fluorescence image of MitroTrakcer Red; G: overlay of E and F; H: overlay of G with bright field image; J: fluorescence image of LysoTracker Red®; K: overlay of I and J; L: overlay of K with bright field image.

**Figure 7:**
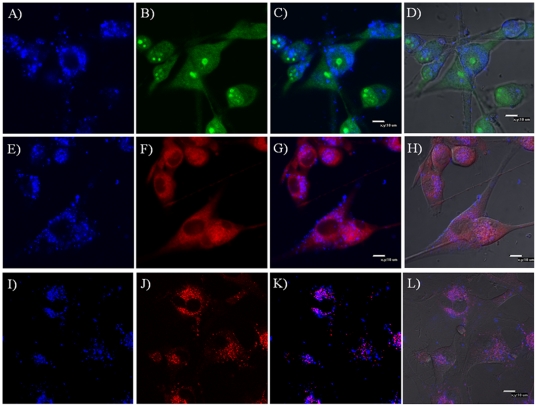
Confocal fluorescence microscopy images of the OF/PCL-*b*-PEG-NH_2_ micelles for U87MG cells. A, E and I: fluorescence images of micelles; B: fluorescence image of SYTO 9; C: overlay of A and B; D: overlay of C with bright field image; F: fluorescence image of MitroTrakcer Red; G: overlay of E and F; H: overlay of G with bright field image; J: fluorescence image of LysoTracker Red®; K: overlay of I and J; L: overlay of K with bright field image.

Bright green emissions with spherical shapes were observed (Figure 6B and Figure 7B), indicating the nucleolus. Around the nucleolus, a boundary between the nuclei and cytoplasm were observed for some cells. For clearer observation, Figure 6B and Figure 7B are magnified and given in supporting materials (Figure S2 and Figure S3). There is almost no overlap of the blue emission with the green emission in the nucleus region, showing that the micelles are not located in nucleus, instead are located in the cytoplasm of the cells (Figure 6C and Figure 7C). Colocalization of micelles with cytoplasmic organelle–selective dyes (LysoTracker Red® and MictoTracker Red) is indicated by pink (Figure 6G, 6K, 7G and 7K). Partial colocalization was observed between blue emissions from **OF** and red emissions from LysoTracker Red® and MitoTracker Red representing acidic organelles (lysosomes and late endosomes) (Figure 6K and 7K) and mitochondria (Figure 6G and 7G) respectively. These results implied that micelles have no specific locations with these organelles, showing random intracellular distributions of the micelles. These observations are in accordance with the studies of other fluorescent micelles [28], [33], [34]. The minimum colocalization of the micelles with the lysosome indicates that micelles had escaped from the acidic compartments of cells. This could possibly be related to the destabilization of the endosomes by the micelles or other cell signaling pathways activated in the cellular compartment to facilitate the micelles' escapes from the endosome/lysosome [33], [35].

**Figure 8:**
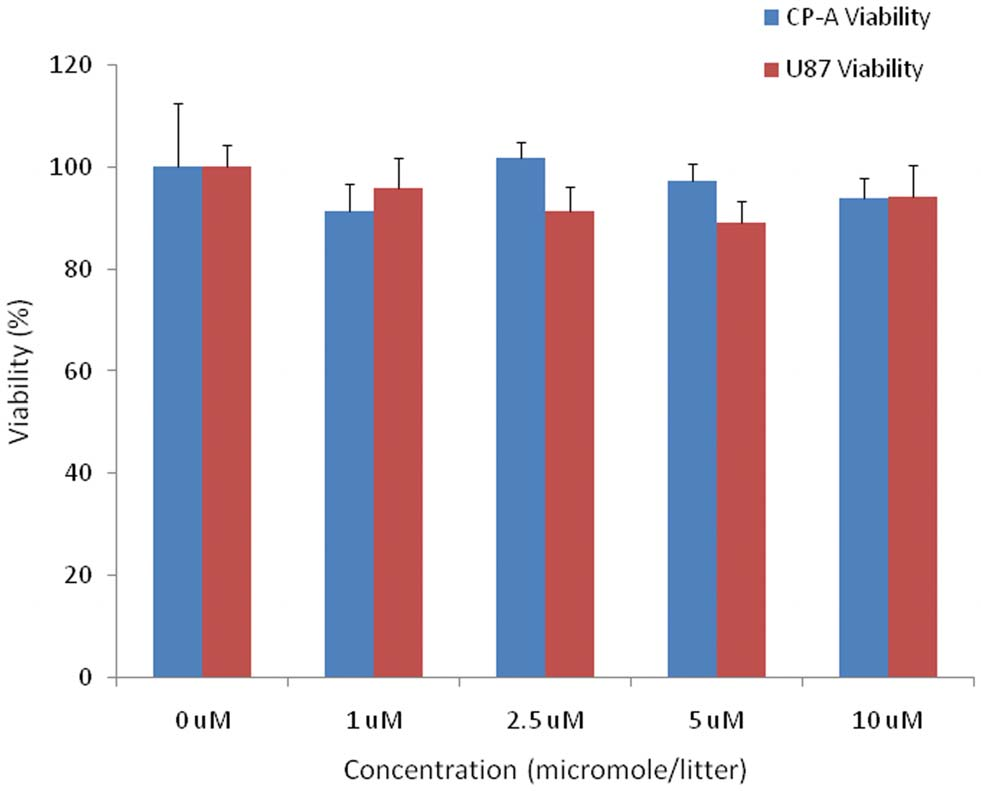
Cell viability of CP-A and U87MG cells at different micelle concentrations. 0 µM indicates the control cells without addition of micelles. **OF** concentrations of 1 to 10 µM correspond to PCL-*b*-PEG-NH_2_ concentrations of 0.025–0.25 mg/mL.

Cytotoxicity of the micelles in the two cell lines was investigated using MTT assay. The MTT assay is based on an intracellular reduction of a tetrazolium dye to a formazan product measured spectrophotometrically and is used for high-throughput screening [36], [37]. Greater than 90% of cells were viable after the cells were stained for 24 hours using OF concentrations of 1–10 µM, which are relevant with the polymer concentration of 0.025–0.25 mg/mL (Figure 8). These observations demonstrated the biocompatibility of the OF-incorporated micelles.

### Conclusion

We have used micelles formed from an amphiphilic PEG-*b*-PCL polymer to incorporate a hydrophobic OF to enable the application of the hydrophobic OF in a biological environment for bioimaging. The micelles were successfully taken up by two different cell lines (U87MG and CP-A). Bioimaging investigation indicates that these micelles were distributed in the cytoplasm area. The micelles were not cytotoxic to U87MG or CP-A cell lines. It is the first time that hydrophobic oligofluorene has been delivered to brain tumor U87MG cells and esophageal precancerous CP-A cells using a PEG-*b*-PCL type of block copolymers. The use of micelles to encapsulate hydrophobic conjugated polymers and to deliver polymers into cells for bioimaging will broaden the applications of conjugated polymers in biological fields. Further investigation of the use of functional conjugated polymer-incorporated micelles for drug delivery is in progress.

## Supporting Information

Figure S1 **Concentration dependent absorption spectra of OF (A) and the absorbance at 380 nm (B).**
(TIF)

Figure S2 **Enlarged** **Figure 6B** **.**
(TIF)

Figure S3 **Enlarged** **Figure 7B** **.**
(TIF)

Text S1 **Determination of OF concentration in micelles.**
(DOC)
