# Supplementary figures and images for: Micelles as Delivery Vehicles for Oligofluorene for Bioimaging
Source: PLoS One. 2011 Sep 6;6(9):e24425. doi: 10.1371/journal.pone.0024425 (PMC3167853; doi:10.1371/journal.pone.0024425)

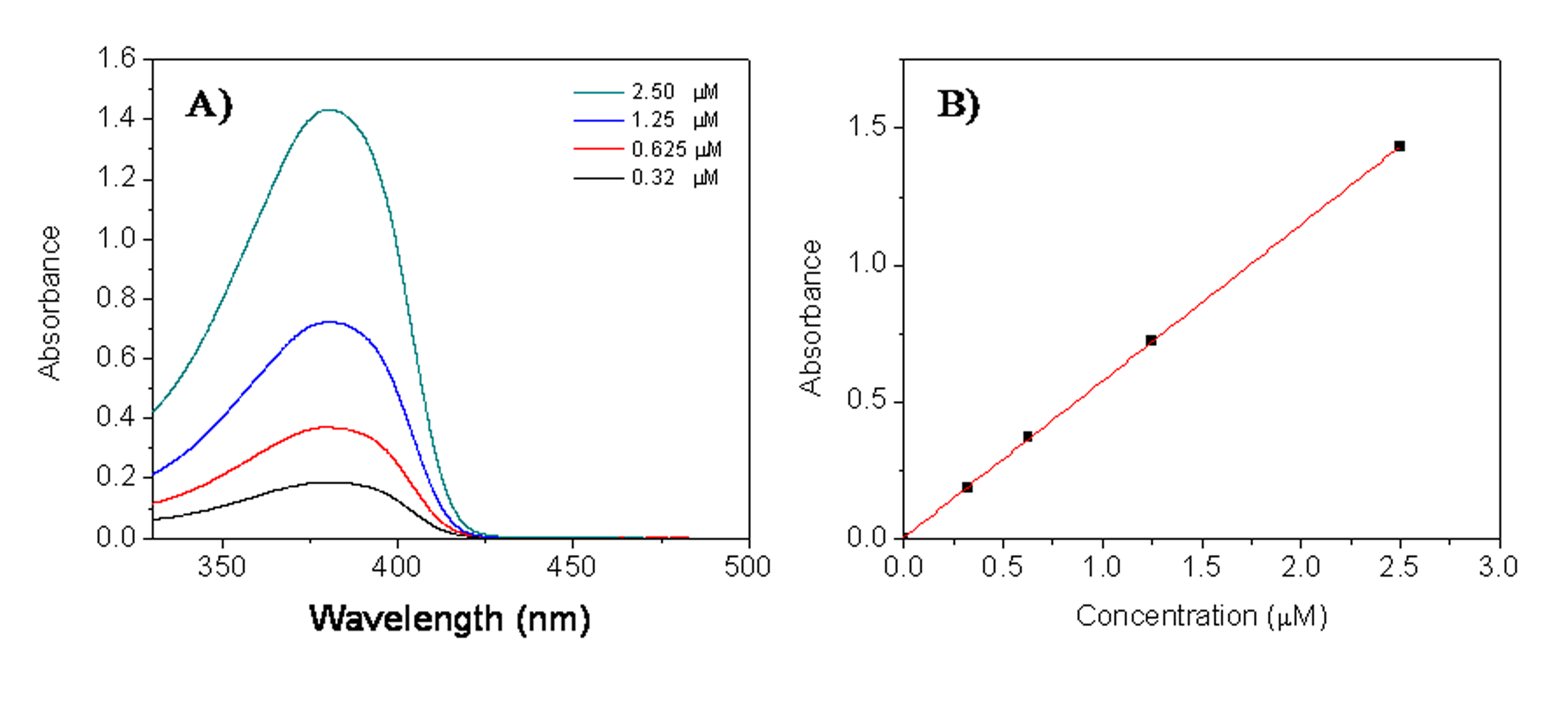

Supplement: Figure S1 — Concentration dependent absorption spectra of OF (A) and the absorbance at 380 nm (B). (TIF) [file pone.0024425.s001.tif]

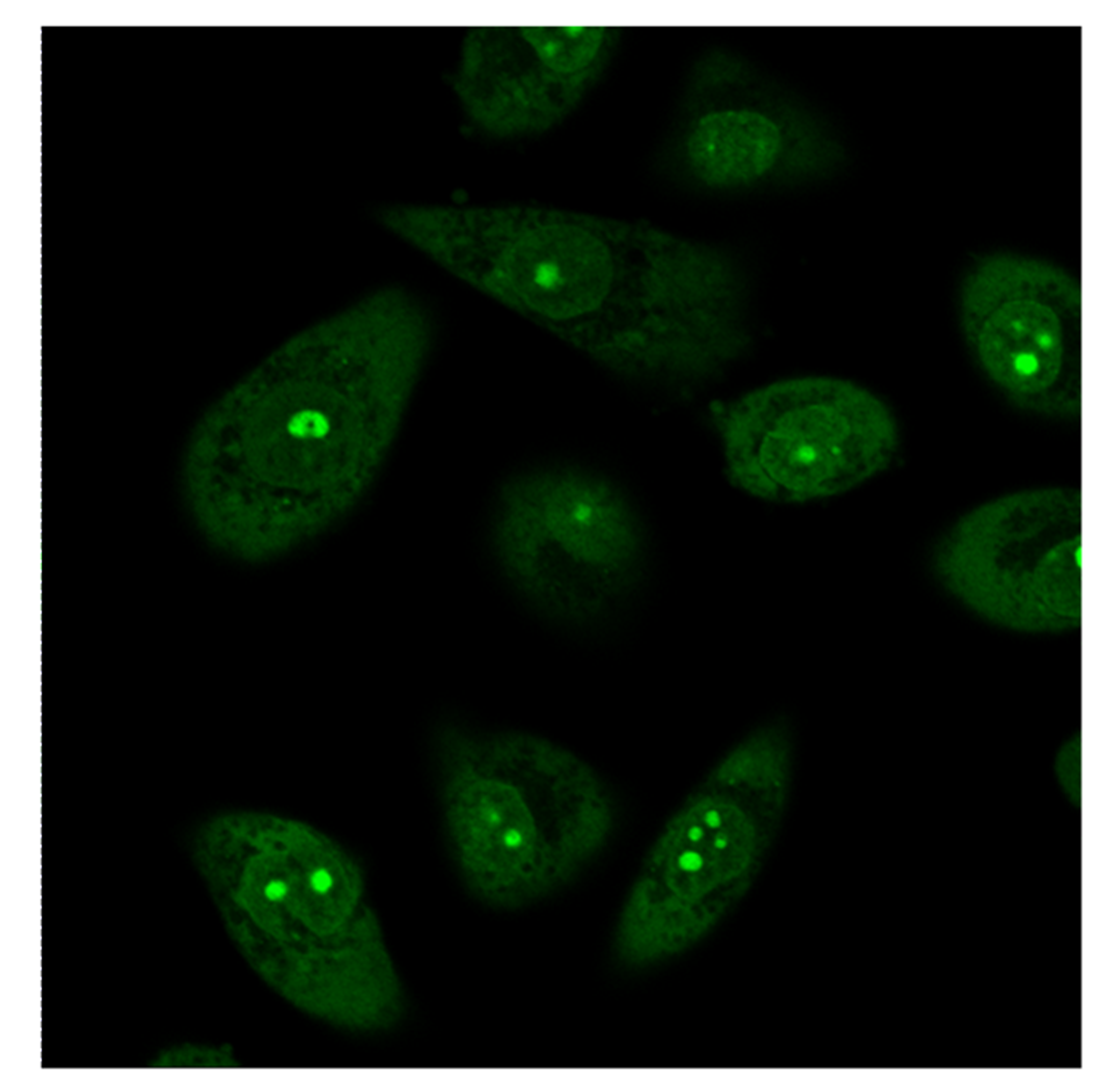

Supplement: Figure S2 — Enlarged Figure 6B . (TIF) [file pone.0024425.s002.tif]

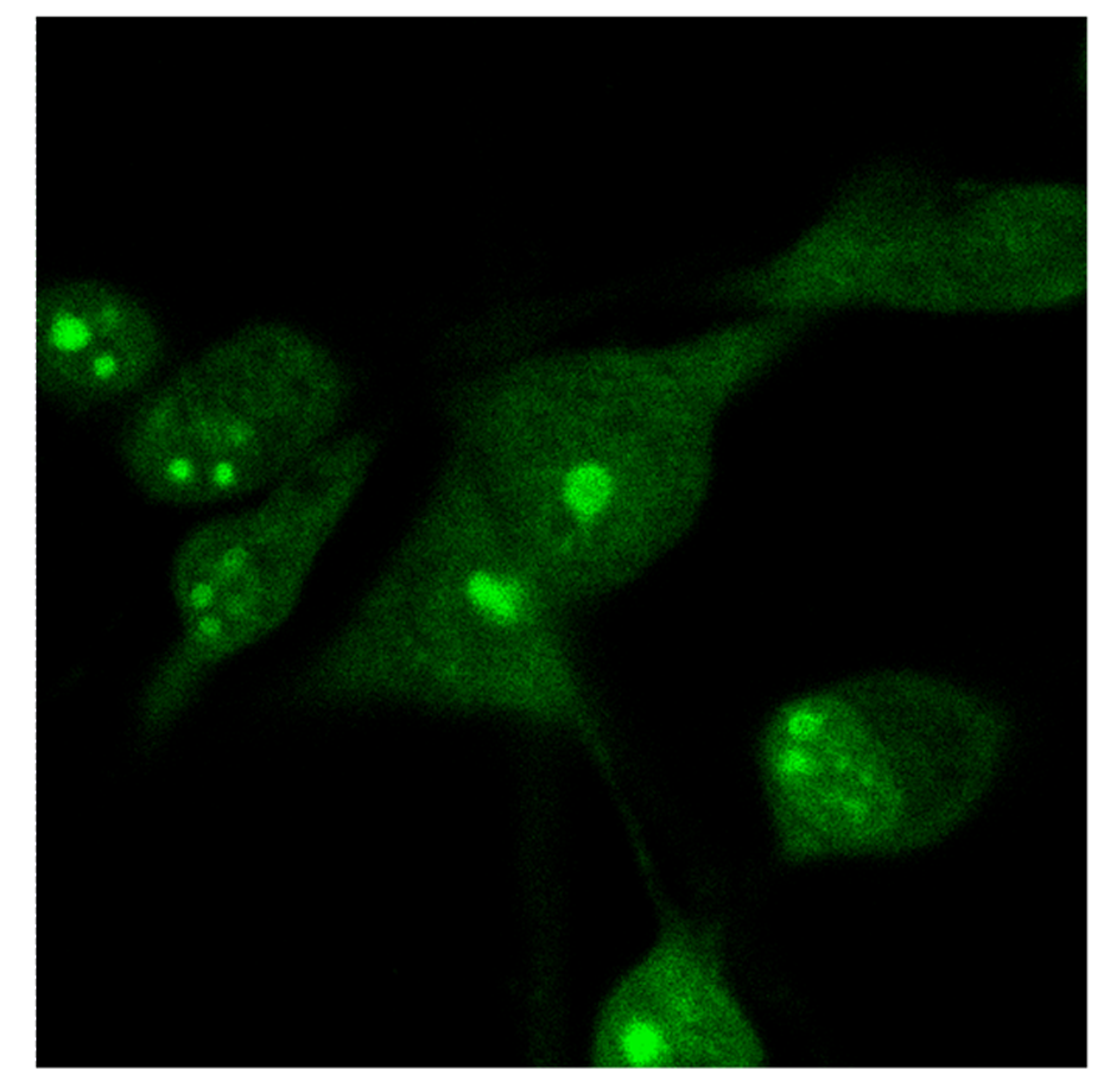

Supplement: Figure S3 — Enlarged Figure 7B . (TIF) [file pone.0024425.s003.tif]
