# Supplementary material for: Micelles as Delivery Vehicles for Oligofluorene for Bioimaging
Source: PLoS One. 2011 Sep 6;6(9):e24425. doi: 10.1371/journal.pone.0024425 (PMC3167853; doi:10.1371/journal.pone.0024425)
Supplement: Text S1 — Determination of OF concentration in micelles. (DOC) [file pone.0024425.s004.doc]

**Text S1 - Determination of OF concentration in micelles.**

**OF** was dissolved in THF to make solutions with concentrations of 0.31, 0.625, 1.25 and 2.5 µM. Typical concentration dependent absorption spectra were given in Figure S3A. Concentration dependent absorbance at 380 nm was plotted in Figure S3B, showing a linear relationship of the polymer concentrations with absorbance, which is used as the calibration cure for concentration determination.

20 µL of **OF** incorporated micelles were freeze dried and then dissolved in 2 mL THF with 100 folds dilution. The THF solution was filtrated using 0.45 µm filter to remove any possible non-dissolved HEPES. The solution was used for absorption measurement. According to the absorbance at 380 nm and the dilution folds, the concentration of **OF** incorporated in micelles was calculated.
